# *Cryptosporidium* and *Giardia* taxa in faecal samples from animals in catchments supplying the city of Melbourne with drinking water (2011 to 2015)

**DOI:** 10.1186/s13071-016-1607-1

**Published:** 2016-06-01

**Authors:** Anson V. Koehler, Shane R. Haydon, Aaron R. Jex, Robin B. Gasser

**Affiliations:** Faculty of Veterinary and Agricultural Sciences, The University of Melbourne, Parkville, Victoria 3010 Australia; Melbourne Water, Docklands, Victoria 3001 Australia; The Walter and Eliza Hall Institute, Parkville, Victoria 3052 Australia

**Keywords:** *Cryptosporidium*, *Giardia*, 60 kDa glycoprotein (*gp60*) gene, Small subunit (*SSU*) of ribosomal RNA gene, Single-strand conformation polymorphism (SSCP), Triose-phosphate isomerase (*tpi*) gene

## Abstract

**Background:**

In a long-term program to monitor pathogens in water catchments serving the City of Melbourne in the State of Victoria in Australia, we detected and genetically characterised *Cryptosporidium* and *Giardia* in faecal samples from various animals in nine water reservoir areas over a period of 4 years (July 2011 to November 2015).

**Methods:**

This work was conducted using PCR-based single-strand conformation polymorphism (SSCP) and phylogenetic analyses of portions of the small subunit of ribosomal RNA (*SSU*) and 60 kDa glycoprotein (*gp60*) genes for *Cryptosporidium*, and triose-phosphate isomerase (*tpi*) gene for *Giardia*.

**Results:**

The prevalence of *Cryptosporidium* was 1.62 % (69 of 4,256 samples); 25 distinct sequence types were defined for p*SSU*, and six for *gp60* which represented *C. hominis* (genotype Ib - subgenotype IbA10G2), *C. cuniculus* (genotype Vb - subgenotypes VbA26, and VbA25), and *C. canis*, *C. fayeri*, *C. macropodum*, *C. parvum, C. ryanae*, *Cryptosporidium* sp. “duck” genotype*, C. suis* and *C. ubiquitum* as well as 12 novel *SSU* sequence types. The prevalence of *Giardia* was 0.31 % (13 of 4,256 samples); all three distinct *tpi* sequence types defined represented assemblage A of *G. duodenalis*.

**Conclusions:**

Of the 34 sequence types (genotypes) characterized here, five and one have been recorded previously for *Cryptosporidium* and *Giardia*, respectively, from humans. Novel genotypes of *Cryptosporidium* and *Giardia* were recorded for *SSU* (*n* = 12), *gp60* (*n* = 4) and *tpi* (*n* = 1); the zoonotic potential of these novel genotypes is presently unknown. Future work will continue to monitor the prevalence of *Cryptosporidium* and *Giardia* genotypes in animals in these catchments, and expand investigations to humans. Nucleotide sequences reported in this paper are available in the GenBank database under accession nos. KU531647–KU531718.

**Electronic supplementary material:**

The online version of this article (doi:10.1186/s13071-016-1607-1) contains supplementary material, which is available to authorized users.

## Background

One of the toughest challenges facing the world’s supply of clean drinking water is contamination from faeces and soil [1, 2]. Diarrhoeal disease is responsible for 10.5 % of deaths in children of less than five years of age [3–5], having a greater impact than malaria and HIV/AIDS combined [5]. Pathogens of concern include viruses, bacteria and protists [1]. Of the latter pathogen group, human-infective taxa (i.e. species and genotypes/assemblages) of *Cryptosporidium* and *Giardia* are highly significant [1, 4–8]. *Cryptosporidium* and *Giardia* are unique in that very small numbers of infective stages (oocysts and cysts, respectively) can cause disease in humans [9, 10] and that these stages are resistant to chlorination and other common water treatments [1, 11]. An example of the tremendous impact these parasites can have was demonstrated in 1993, with a major outbreak of cryptosporidiosis in Milwaukee, USA [12], which affected more than 400,000 people and resulted in 100 deaths. This case emphasizes the major public health importance of waterborne diseases and the need for their sustained prevention.

Melbourne (Victoria, Australia; population ~4 million) is one of the few cities in the world that receives largely unfiltered drinking water from protected wilderness catchment areas. The management of Melbourne’s ten main water catchment areas includes restricted access for humans, long water retention times and an intense program of testing and monitoring for pathogens in source water. These catchments represent habitat for native and feral animals, such that the monitoring of zoonotic pathogens is central to management and the prevention of outbreaks of waterborne disease. In 2008, we initiated a program to monitor *Cryptosporidium* and *Giardia* in faecal samples from various mammals and birds in the Melbourne’s catchments [13]. To do this, we collected 2,009 fresh faecal samples (from June 2009 to June 2011) and tested them using an established and validated PCR-based mutation scanning-coupled sequencing approach (cf. [14–16]), combined with phylogenetic analyses of loci (*SSU* and *gp60*) in the small subunit (*SSU*) of ribosomal RNA and 60-kDa glycoprotein (*gp60*) genes to detect and characterise *Cryptosporidium*, and another locus (*tpi*) in the triose-phosphate isomerase (*tpi*) gene to identify and classify *Giardia* [13]. In total, *Cryptosporidium* and *Giardia* were detected in 2.8 and 3.4 % of all 2009 samples tested, respectively, and 35 previously undescribed genotypes were reported [13]. In spite of this relatively low prevalence, the findings from this study emphasized a need for a sustained program.

Therefore, from July 2011 to November 2015, we extended our monitoring program, and genetically characterised *Cryptosporidium* and *Giardia* from native and introduced animals in Melbourne’s water catchments, in order to continually assess the prevalence and diversity of *Cryptosporidium* and *Giardia* taxa, evaluate their host affiliations, geographical distributions and zoonotic potential, and support catchment management. In the present article, we describe the results from this 4-year study and discuss the findings in a water industry context.

## Methods

### Melbourne’s catchments

**Fig. 1:**
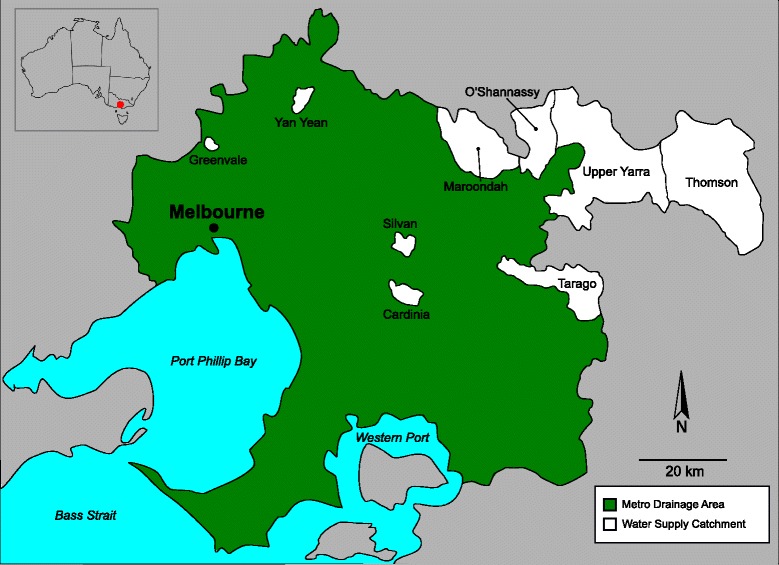
Map of the Melbourne Water catchment areas from where the 4,256 faecal samples from animals were collected

Greater Melbourne sources its municipal drinking water from ten main water catchment reservoirs. Approximately 80 % of Melbourne’s drinking water is drawn from ‘closed’ catchments in the Yarra Ranges (~85 km east of Melbourne), which cover 157,000 hectares of eucalypt forest, with restricted human and domestic animal access, to minimise the risk of waterborne diseases. The remaining 20 % of Melbourne’s water comes from ‘open’ catchments, in which some farming and human activities are permitted. All water undergoes treatment in accordance with national and international guidelines [17, 18]. The nine reservoirs studied here (Fig. 1) are located north and east of Melbourne’s central business district (CBD), are less than 90 km apart and include: Cardinia (CA) 37°47'S, 145°24'E; Greenvale (GV) 37°37'S, 144°54'E; Maroondah (MR) 37°38'S, 145°33'E; O’Shannassy (OS) 37°40'S, 145°48'E; Silvan (SV) 37°50'S, 145°25’E; Tarago (TAR) 37°59'S, 145°55’E; Thompson (TH) 37°47'S, 146°21'E; Upper Yarra (UY) 37°40'S, 145°55'E; and Yan Yean (YY) 37°33'S, 145°08'E. Reservoirs MR, OS, TH and UY are situated in the densely forested Yarra Ranges catchment, whereas YY reservoir is a much smaller catchment north of the CBD and surrounded by residential and grazing land. The remaining reservoirs, including CA, GV and SV, act as storage facilities for the larger catchments and have eucalypt and/or pine forests. TAR is the one ‘open’ water supply catchment, which permits farming in the land surrounding the reservoir. All regions have small areas of grassland adjacent to water reservoirs, and it is here that faecal samples were collected.

### Samples and isolation of genomic DNA

A total of 4,256 faecal deposits from *Canis familiaris* (dog), *Vulpes vulpes* (fox), *Felis catus* (cat), *Dromaius novaehollandiae* (emu), *Wallabia bicolor* (swamp wallaby), *Macropus giganteus* (Eastern grey kangaroo), *Trichosurus vulpecula* (common brushtail possum), *Oryctolagus cuniculus* (rabbit), *Rattus lutreolus* (swamp rat), *Rattus fuscipes* (bush rat) and *Vombatus ursinus* (common wombat), together with waterbirds, principally the Australian wood duck (*Chenonetta jubata*), and deer, including *Rusa unicolour* (sambar)*, Cervus elaphus* (red) and *Dama dama* (fallow), and samples of unknown host origin were collected from nine locations from July 2011 to November 2015 (see Additional file 1: Table S1). Specifically, samples were collected from CA (*n* = 718), GV (*n* = 638), MR (*n* = 344), OS (*n* = 622); SV (*n* = 527), TAR (*n* = 61), TH (*n* = 31), UY (*n* = 714) and YY (*n* = 601). Scats were identified using a field guide [19], and host identity was confirmed, as required, by PCR-based sequencing of a region of the mitochondrial cytochrome *b* gene from faecal DNA using a similar approach to that described by Dalen et al. [20]. Genomic DNA was extracted directly from 0.25 g of faeces using the PowerSoil kit (MoBio, USA), according to the manufacturer’s instructions.

### Polymerase chain reaction (PCR) amplification of genetic loci

Genomic DNA samples were subjected to nested PCR-based analyses of three loci. For the specific identification of *Cryptosporidium*, a portion of the *SSU* gene (~240 bp) was used [13], and genotypic/subgenotypic classification was achieved employing part of the *gp60* gene (250–350 bp) [13]. For the genetic characterisation of *Giardia* (to the level of assemblage), a portion of the *tpi* gene (~530 bp) was employed [21]. PCR was carried out in a volume of 50 μl containing 10 mM Tris-HCl (pH 8.4), 50 mM KCl (Promega, Madison, USA), 2.0–3.0 mM of MgCl_2_ (depending on the locus), 200 μM of each deoxynucleotide triphosphate, 50 pmol of each primer and 1 U of either Go*Taq* (Promega) or Mango*Taq*™ (Bioline, USA) DNA polymerase.

For *Cryptosporidium*, primary amplification of *SSU* was achieved using primers XF2 (forward: 5'-GGA AGG GTT GTA TTT ATT AGA TAA AG-3') and XR2 (reverse: 5'-AAG GAG TAA GGA ACA ACC TCC A-3') [22], followed by nested amplification of *SSU* using the internal primers pSSUf (forward: 5'-AAA GCT CGT AGT TGG ATT TCT GTT-3') and pSSUr (reverse: 5'-ACC TCT GAC TGT TAA ATA CRA ATG C-3') [23]. For primary amplification, a cycling protocol of 94 °C for 5 min (initial denaturation), followed by 30 cycles of 94 °C for 45 s (denaturation), 45 °C for 2 min (annealing) and 72 °C for 1.5 min (extension), with a final extension of 72 °C for 10 min was employed. Secondary amplification was achieved employing 94 °C for 5 min, followed by 35 cycles of 94 °C for 30 s, 55 °C for 30 s, and 72 °C for 30 s, with a final extension of 72 °C for 10 min.

For selected samples, *Cryptosporidium* was further characterized using a longer region (~590 bp) of the *SSU* gene. This region was first PCR-amplified employing primers 18SiCF2 (forward: 5'-GAC ATA TCA TTC AAG TTT CTG ACC-3') and 18SiCR2 (reverse: 5'-CTG AAG GAG TAA GGA ACA ACC-3'), followed by a nested amplification using primers 18SiCF1 (forward: 5'-CCT ATC AGC TTT AGA CGG TAG G-3') and 18SiCR1 (reverse: 5'-TCT AAG AAT TTC ACC TCT GAC TG-3') [24]. Both amplifications utilized the cycling protocol: 94 ° for 5 min (initial denaturation), followed by 45 cycles of 94 °C for 30 s (denaturation), 58 °C for 30 s (annealing) and 72 °C for 30 s (extension), with a final extension of 72 °C for 10 min.

The *gp60* gene (~1 kb) was first amplified using primers gp15-ATG (forward: 5'-ATG AGA TTG TCG CCT CAT TAT C-3') and gp15-STOP (reverse: 5'-TTA CAA CAC GAA TAA GGC TGC-3') [25], followed by the nested amplification of *gp60* using primers gp15-15A (forward: 5'-GCC GTT CCA CTC AGA GGA AC-3') and gp15-15E (reverse: 5'-CCA CAT TAC AAA TGA AGT GCC GC-3') [26]. Primary amplification of *gp60* utilised the cycling protocol, 94 °C for 5 min (initial denaturation), followed by 35 cycles of 94 °C for 30 s (denaturation), 55 °C for 45 s (annealing) and 72 °C for 1 min (extension), with a final extension of 72 °C for 10 min. For the amplification of *gp60*, we employed 94 °C for 5 min, followed by 30 cycles of 94 °C for 30 s, 55 °C for 30 s, and 72 °C for 30 s, with a final extension at 72 °C for 10 min.

For some samples, *Cryptosporidium* was further characterized using a longer region (~850 bp) of the *gp60* gene. This region was PCR-amplified using primers AL3531 (forward: 5'-ATA GTC TCC GCT GTA TTC-3') and AL3535 (reverse: 5'-GGA AGG AAC GAT GTA TCT-3'), followed by a nested amplification using primers AL3532 (forward: 5'-TCC GCT GTA TTC TCA GCC-3') and AL3534 (reverse: 5'-GCA GAG GAA CCA GCA TC-3') [27]. For both amplifications, the following cycling protocol was used: 94 °C for 5 min (initial denaturation), followed by 35 cycles of 94 °C for 45 s (denaturation), 50 °C for 45 s (annealing) and 72 °C for 60 s (extension), with a final extension of 72 °C for 10 min.

For *Giardia*, the *tpi* locus was amplified using primers AL3543 (forward: 5'-AAA TTA TGC CTG CTC GTC G-3') and AL3546 (reverse: 5'-CAA ACC TTT TCC GCA AAC C-3’), followed by the nested amplification of *tpi* employing primers AL3544 (forward: 5'-CCC TTC ATC GGT GGT AAC TT-3') and AL3545 (reverse: 5'-GTG GCC ACC ACT CCC GTG CC-3') [21]. For the primary amplification, the cycling protocol was 94 °C for 5 min (initial denaturation), followed by 35 cycles of 94 °C for 45 s (denaturation), 50 °C for 45 s (annealing), and 72 °C for 1 min (extension) and a final extension of 72 °C for 10 min. Secondary amplification of *tpi* was achieved employing 94 °C for 5 min, followed by 35 cycles of 94 °C for 45 s, 55 °C for 30 s, and 72 °C for 1 min, with a final extension at 72 °C for 10 min.

### Mutation scanning, sequencing and phylogenetic analyses

Single-strand conformation polymorphism (SSCP) analysis was used to scan for sequence variation within and among *SSU* and *gp60* amplicons (e.g. [13, 16]). In brief, 1 μl of each secondary amplicon (< 450 bp) was mixed with 5 μl of DNA sequencing-stop solution (Promega) and 5 μl of H_2_0, heat-denatured at 94 °C/30 min, snap-cooled on a freeze-block (-20 °C) and then subjected to electrophoresis at 74 V at 7.4 °C (constant) for 16 h in a GMA Wide Mini S-2x25 gel in a SEA 2000 rig (Elchrom Scientific AG) using TAE buffer (40 mM Tris base, 20 mM acetic acid, 1.0 mM EDTA, Bio-Rad, USA). A control sample (representing a known genotype) was included on each gel to ensure the reproducibility of profiles representing this sample among gels.

Following SSCP-based analysis, selected amplicons representing each distinct electrophoretic profile and all *SSU*, *gp60* (*Cryptosporidium*) and *tpi* (*Giardia*) amplicons were treated with shrimp alkaline phosphatase and exonuclease I (ThermoFisher, Waltham, USA), according to the manufacturer’s instructions, and then subjected to bi-directional automated sequencing (BigDye® Terminator v.3.1 chemistry, Applied Biosystems, USA) using the same primers employed in the secondary PCR. Sequence quality was verified by comparison with corresponding electropherograms using the program Geneious v.8 [28]. Sequences were aligned using the program MUSCLE [29], and alignments were adjusted manually using the program Mesquite v.2.75 [30]. Sequences were then compared with those available in the GenBank database using BLASTn.

Phylogenetic analysis of sequence data was conducted by Bayesian inference (BI) using Monte Carlo Markov Chain (MCMC) analysis in MrBayes v.3.2.3 [31]. The likelihood parameters set for BI analysis of *SSU* data were based on the Akaike Information Criteria (AIC) test in jModeltest v.2.1.7 [32]. For *SSU* (*Cryptosporidium*) and *tpi* (*Giardia*) data, the number of substitutions (Nst) was set at 6, with a gamma-distribution and a proportion of invariable sites. For the separate analyses of *gp60* (*Cryptosporidium*) sequence data, the Nst was set at 6, with an equal rate among sites. Posterior probability (pp) values were calculated by running 2,000,000 generations with four simultaneous tree-building chains. Trees were saved every 100th generation. At the end of each run, the standard deviation of split frequencies was < 0.01, and the potential scale reduction factor approached one. A 50 % majority rule consensus tree for each analysis was constructed based on the final 75 % of trees generated by BI. Analyses were run three times to ensure convergence and insensitivity to priors. Outgroups used in the analyses were *Giardia muris* for *G. duodenalis* (*tpi*), *C. hominis* for *gp60* and *C. muris* for *SSU*.

## Results

### Molecular detection of *Cryptosporidium*, and taxon identity based on *SSU*

**Fig. 2:**
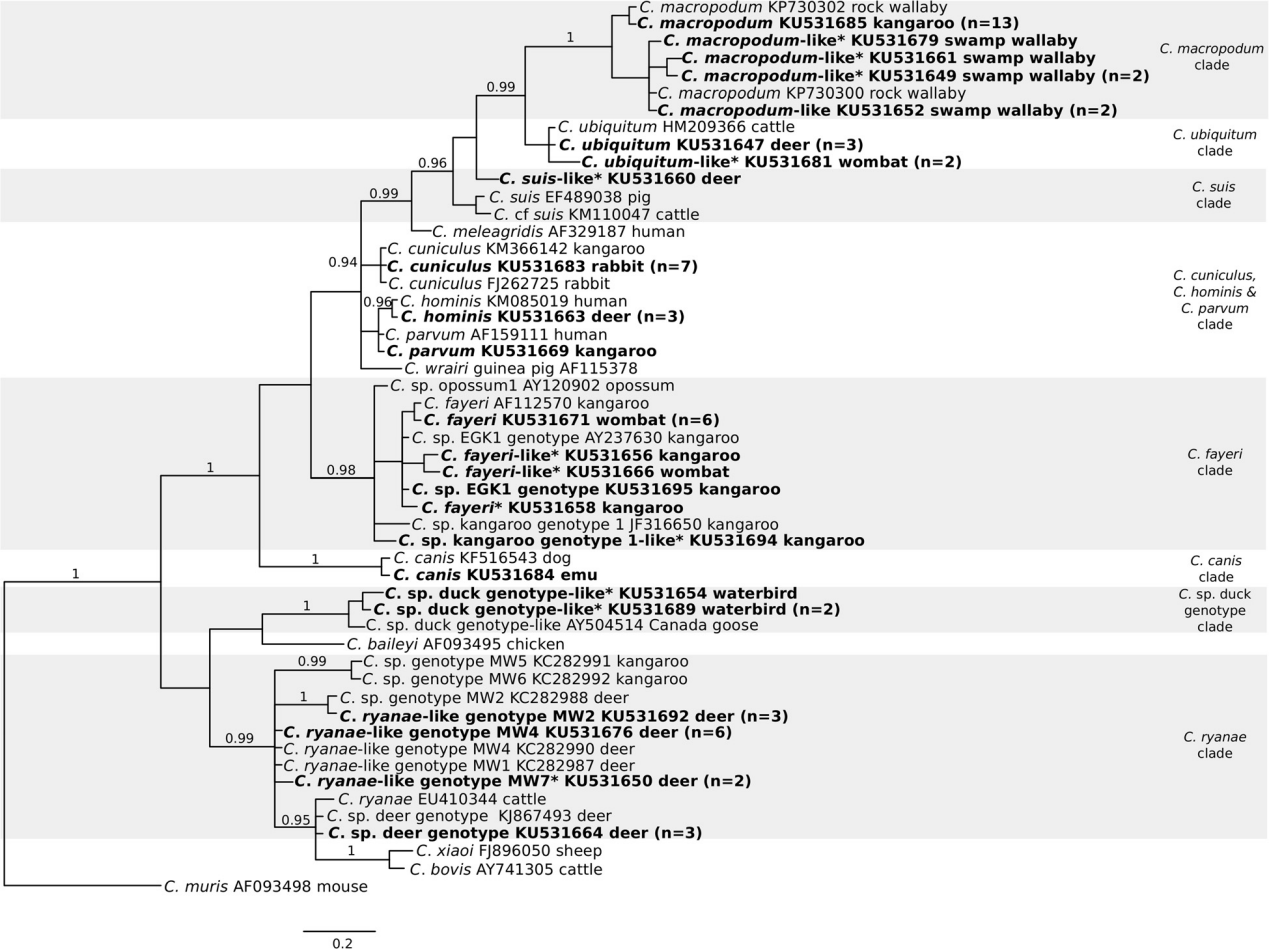
Relationships among *Cryptosporidium* taxa inferred from the phylogenetic analysis of partial small subunit ribosomal RNA gene (*SSU*) sequence data by Bayesian inference (BI). Posterior probabilities are indicated at all major nodes. Bold-type indicates *Cryptosporidium* species or genotypes characterized from faecal DNA samples in this study. In parentheses are the numbers of samples representing a particular species, genotype and sequence (GenBank accession numbers indicated). Novel genotypes (*). Scale-bar represents the number of substitutions per site

We conducted mutation scanning and sequence analyses of all amplicons (*n* = 69) produced from 4,256 (1.62 %) faecal DNA samples and identified them to species and/or genotype of *Cryptosporidium*. A total of 64 samples were characterised by their *SSU* sequences; 52 were assigned GenBank accession nos. (KU531647–KU531698; Tables 1 and 2), of which 24 sequences were selected as representatives for phylogenetic analysis (Fig. 2; Additional file 2: Table S4). In total, there were 12 novel sequences (i.e. < 100 % identity with a sequence on GenBank) for *SSU*. Samples that were test-positive for *SSU* were assessed according to catchment (Additional file 1: Table S2) and host (Additional file 1: Table S3). Overall, prevalence was assessed by catchment in Table 2.

**Table 1:** Summary of epidemiological and molecular information pertaining to the pathogen test-positive faecal samples collected from the Melbourne Water catchments (July 2011 to November 2015)

Sample code	Host	Locality	Date	Pathogen	Typing	Method	GenBank accession no.
MR4158	Wombat	Maroondah	12-Dec-13	*Cryptosporidium fayeri*	*SSU*	Sequencing	KU531671
MR4198	Wombat	Maroondah	12-Dec-13	*Cryptosporidium fayeri*	*SSU*	SSCP	KU531671^a^
MR4199	Wombat	Maroondah	12-Dec-13	*Cryptosporidium fayeri*	*SSU*	SSCP	KU531671^a^
MR4200	Wombat	Maroondah	12-Dec-13	*Cryptosporidium fayeri*	*SSU*	SSCP	KU531671^a^
MR4211	Wombat	Maroondah	12-Dec-13	*Cryptosporidium fayeri*	*SSU*	SSCP	KU531671^a^
MR4231	Wombat	Maroondah	12-Dec-13	*Cryptosporidium fayeri*	*SSU*	Sequencing	KU531672
GV3073	Kangaroo	Greenvale	16-Oct-12	***Cryptosporidium fayeri-*** **like**	*SSU*	Sequencing	KU531656
YY3126	Kangaroo	Yan Yean	16-Oct-12	***Cryptosporidium fayeri*** **-like**	*SSU*	Sequencing	KU531658
C3616	Wombat	Cardinia	31-Jul-13	***Cryptosporidium fayeri*** **-like**	*SSU*	Sequencing	KU531666
YY6091	Kangaroo	Yan Yean	09-Jul-15	*Cryptosporidium* sp. EGK1 genotype (C. *fayeri*-like)	*SSU*	Sequencing	KU531695
YY6016	Kangaroo	Yan Yean	09-Jul-15	***Cryptosporidium*** **sp. Kangaroo genotype I (** ***C. fayeri*** **-like)**	*SSU*	Sequencing	KU531694
OS2785	Deer	O'Shannassy	Jul-12	*Cryptosporidium hominis*	*SSU*	Sequencing	na
UY3513	Deer	Upper Yarra	25-Jun-13	*Cryptosporidium hominis*	*SSU*	Sequencing	KU531663
MR3443	Wallaby	Maroondah	09-May-13	*Cryptosporidium hominis* (IbA10G2)	*gp60*	Sequencing	KU531699
GV3952	Kangaroo	Greenvale	20-Aug-13	*Cryptosporidium parvum*	*SSU*	Sequencing	KU531669
SV5306	Rabbit	Silvan	26-Nov-14	*Cryptosporidium cuniculus*	*SSU*	Sequencing	KU531683
GV6100	Rabbit	Greenvale	09-Jul-15	***Cryptosporidium cuniculus*** **(VbA24)**	*SSU*	SSCP	KU531697^a^
GV6100	Rabbit	Greenvale	09-Jul-15	***Cryptosporidium cuniculus*** **(VbA24)**	*gp60*	Sequencing	KU531704
SV5945	Rabbit	Silvan	21-May-15	***Cryptosporidium cuniculus*** **(VbA25)**	*SSU*	Sequencing	KU531693
SV5945	Rabbit	Silvan	21-May-15	***Cryptosporidium cuniculus*** **(VbA25)**	*gp60*	Sequencing	KU531702
GV6098	Rabbit	Greenvale	09-Jul-15	***Cryptosporidium cuniculus*** **(VbA25)**	*SSU*	Sequencing	KU531696
GV6098	Rabbit	Greenvale	09-Jul-15	***Cryptosporidium cuniculus*** **(VbA25)**	*gp60*	Sequencing	KU531703
YY3790	Kangaroo	Yan Yean	20-Aug-13	*Cryptosporidium cuniculus* (VbA26)	*gp60*	Sequencing	KM366140
YY3790	Kangaroo	Yan Yean	20-Aug-13	*Cryptosporidium cuniculus* (VbA26)	*SSU*	Sequencing	KM366142
YY3809	Kangaroo	Yan Yean	20-Aug-13	*Cryptosporidium cuniculus* (VbA26)	*gp60*	Sequencing	KU531700
GV5010	Rabbit	Greenvale	04-Sep-14	*Cryptosporidium cuniculus* (VbA26)	*gp60*	Sequencing	KU531701
GV6131	Rabbit	Greenvale	09-Jul-15	*Cryptosporidium cuniculus* (VbA26)	*gp60*	SSCP	KU531705^a^
GV6132	Rabbit	Greenvale	09-Jul-15	*Cryptosporidium cuniculus* (VbA26)	*gp60*	SSCP	KU531705^a^
GV6137	Rabbit	Greenvale	09-Jul-15	***Cryptosporidium cuniculus*** **(VbA26)**	*SSU*	Sequencing	KU531697
GV6137	Rabbit	Greenvale	09-Jul-15	***Cryptosporidium cuniculus*** **(VbA26)**	*gp60*	Sequencing	KU531705
C5371	Emu	Cardinia	08-Jan-15	*Cryptosporidium canis*	*SSU*	Sequencing	KU531684
OS3311	Deer	O'Shannassay	08-Apr-13	***Cryptosporidium suis*** **-like**	*SSU*	Sequencing	KU531660
C2202	Deer	Cardinia	Aug-11	*Cryptosporidium ubiquitum*	*SSU*	Sequencing	KU531647
OS5301	Deer	O'Shannassay	26-Nov-14	*Cryptosporidium ubiquitum*	*SSU*	Sequencing	KU531682
OS6339	Deer	O'Shannassay	20-Nov-15	*Cryptosporidium ubiquitum*	*SSU*	Sequencing	KU531698
C3604	Wombat	Cardinia	31-Jul-13	***Cryptosporidium ubiquitum*** **-like**	*SSU*	Sequencing	KU531665
OS5267	Wombat	O'Shannassay	26-Nov-14	***Cryptosporidium ubiquitum*** **-like**	*SSU*	Sequencing	KU531681
GV3044	Kangaroo	Greenvale	16-Oct-12	*Cryptosporidium macropodum*	*SSU*	Sequencing	KU531655
GV4434	Kangaroo	Greenvale	14-Mar-14	*Cryptosporidium macropodum*	*SSU*	Sequencing	KU531673
GV4441	Kangaroo	Greenvale	14-Mar-14	*Cryptosporidium macropodum*	*SSU*	Sequencing	KU531674
GV4992	Kangaroo	Greenvale	04-Sep-14	*Cryptosporidium macropodum*	*SSU*	Sequencing	KU531677
GV4994	Kangaroo	Greenvale	04-Sep-14	*Cryptosporidium macropodum*	*SSU*	SSCP	KU531677^a^
GV5000	Kangaroo	Greenvale	04-Sep-14	*Cryptosporidium macropodum*	*SSU*	SSCP	KU531677^a^
YY5091	Kangaroo	Yan Yean	04-Sep-14	*Cryptosporidium macropodum*	*SSU*	Sequencing	KU531678
GV5505	Kangaroo	Greenvale	20-Feb-15	*Cryptosporidium macropodum*	*SSU*	Sequencing	KU531685
GV5543	Kangaroo	Greenvale	20-Feb-15	*Cryptosporidium macropodum*	*SSU*	Sequencing	KU531686
GV5552	Kangaroo	Greenvale	20-Feb-15	*Cryptosporidium macropodum*	*SSU*	Sequencing	KU531687
GV5558	Kangaroo	Greenvale	20-Feb-15	*Cryptosporidium macropodum*	*SSU*	SSCP	KU531688^a^
GV5563	Kangaroo	Greenvale	20-Feb-15	*Cryptosporidium macropodum*	*SSU*	SSCP	KU531688^a^
GV5573	Kangaroo	Greenvale	20-Feb-15	*Cryptosporidium macropodum*	*SSU*	Sequencing	KU531688
OS2816	Wallaby	O'Shannassy	Jul-12	***Cryptosporidium macropodum*** **-like**	*SSU*	Sequencing	KU531649
OS2827	Wallaby	O'Shannassy	Jul-12	*Cryptosporidium macropodum*-like	*SSU*	Sequencing	KU531652
SV3188	Wallaby	Silvan	19-Nov-12	*Cryptosporidium macropodum*-like	*SSU*	Sequencing	KU531659
OS3365	Wallaby	O'Shannassay	08-Apr-13	***Cryptosporidium macropodum*** **-like**	*SSU*	Sequencing	KU531661
C3623	Wallaby	Cardinia	31-Jul-13	*Cryptosporidium macropodum*-like	*SSU*	Sequencing	KU531667
OS5235	Wallaby	O'Shannassay	26-Nov-14	***Cryptosporidium macropodum*** **-like**	*SSU*	Sequencing	KU531679
UY5645	Waterbird	Upper Yarra	30-Mar-15	***Cryptosporidium*** **sp. duck genotype-like**	*SSU*	Sequencing	KU531689
UY5649	Waterbird	Upper Yarra	30-Mar-15	***Cryptosporidium*** **sp. duck genotype-like**	*SSU*	SSCP	KU531689^a^
UY2975	Waterbird	Upper Yarra	30-Aug-12	***Cryptosporidium*** **sp. duck-like genotype**	*SSU*	Sequencing	KU531654
OS4106	Deer	O'Shannassay	19-Nov-13	*Cryptosporidium ryanae-*like MW2	*SSU*	Sequencing	KU531670
OS5242	Deer	O'Shannassay	26-Nov-14	*Cryptosporidium ryanae-*like MW2	*SSU*	Sequencing	KU531680
C5875	Deer	Cardinia	21-May-15	*Cryptosporidium ryanae-*like MW2	*SSU*	Sequencing	KU531692
OS2316	Deer	O'Shannassy	Dec-11	*Cryptosporidium ryanae-*like MW4	*SSU*	Sequencing	KU531648
YY3874	Deer	Yan Yean	20-Aug-13	*Cryptosporidium ryanae-*like MW4	*SSU*	Sequencing	KU531668
OS4606	Deer	O'Shannassay	14-Apr-14	*Cryptosporidium ryanae-*like MW4	*SSU*	Sequencing	KU531675
C4873	Deer	Cardinia	24-Jul-14	*Cryptosporidium ryanae-*like MW4	*SSU*	Sequencing	KU531676
UY5700	Deer	Upper Yarra	30-Mar-15	*Cryptosporidium ryanae-*like MW4	*SSU*	Sequencing	KU531690
C5846	Deer	Cardinia	21-May-15	*Cryptosporidium ryanae-*like MW4	*SSU*	Sequencing	KU531691
OS2821	Deer	O'Shannassy	Jul-12	***Cryptosporidium ryanae-*** **like MW7**	*SSU*	Sequencing	KU531650
OS2822	Deer	O'Shannassy	Jul-12	***Cryptosporidium ryanae-*** **like MW7**	*SSU*	Sequencing	KU531651
MR3424	Deer	Maroondah	09-May-13	***Cryptosporidium ryanae-*** **like MW7**	*SSU*	Sequencing	KU531662
UY2900	Deer	Upper Yarra	30-Aug-12	*Cryptosporidium* sp. deer genotype	*SSU*	Sequencing	KU531653
YY3101	Deer	Yan Yean	16-Oct-12	*Cryptosporidium* sp. deer genotype	*SSU*	Sequencing	KU531657
UY3518	Deer	Upper Yarra	25-Jun-13	*Cryptosporidium* sp. deer genotype	*SSU*	Sequencing	KU531664
TH2278	Rabbit	Thomson	25-Sep-11	*Giardia duodenalis* AI	*tpi*	Sequencing	KU531708
TH2291	Deer	Thomson	25-Sep-11	***Giardia duodenalis*** **AI**	*tpi*	Sequencing	KU531709
SV2382	Kangaroo	Silvan	2-Dec-11	*Giardia duodenalis* AI	*tpi*	Sequencing	KU531710
MR4752	Wombat	Maroondah	18-Jun-14	*Giardia duodenalis* AI	*tpi*	Sequencing	KU531718
TAR2129	Deer	Tarago	07-Jul-11	*Giardia duodenalis* AIII	*tpi*	Sequencing	KU531706
TAR2135	Deer	Tarago	07-Jul-11	*Giardia duodenalis* AIII	*tpi*	Sequencing	KU531707
OS4115	Deer	O'Shannassy	19-Nov-13	*Giardia duodenalis* AIII	*tpi*	Sequencing	KU531711
OS4135	Deer	O'Shannassy	19-Nov-13	*Giardia duodenalis* AIII	*tpi*	Sequencing	KU531712
UY4624	Deer	Upper Yarra	20-May-14	*Giardia duodenalis* AIII	*tpi*	Sequencing	KU531713
UY4634	Deer	Upper Yarra	20-May-14	*Giardia duodenalis* AIII	*tpi*	Sequencing	KU531714
UY4635	Deer	Upper Yarra	20-May-14	*Giardia duodenalis* AIII	*tpi*	Sequencing	KU531715
UY4638	Deer	Upper Yarra	20-May-14	*Giardia duodenalis* AIII	*tpi*	Sequencing	KU531716
UY4661	Deer	Upper Yarra	20-May-14	*Giardia duodenalis* AIII	*tpi*	Sequencing	KU531717

Bold-type indicates a novel genotype. na: not available; length of the sequence determined (< 200 bp) was less than that required to be assigned a GenBank accession number; sequence available from authors. ^a^ indicates accession number represented by an SSCP profile

**Table 2:** The total numbers of each host sampled in each catchment, as part of the Melbourne Water Corporation sampling program for waterborne pathogens (July 2011 to November 2015), together with the numbers of animals PCR test-positive for species/genotypes of *Cryptosporidium* (number of test-positive samples)

Catchment	Emu	Waterbird	Deer	Rabbit	Kangaroo	Wallaby	Wombat	Total	Prevalence (%)
Cardinia	1 *C. canis*		4 (3 *C. ryanae*, **1** ***C. ubiquitum***)			1 *C. macropodum*	2 (1 *C. fayeri*, **1** ***C. ubiquitum***)	718	1.11
Greenvale				**6** ***C. cuniculus***	14 (12 *C. macropodum*, 1. *C. fayeri*, **1** ***C. parvum***)			638	3.13
Maroondah			1 *C. ryanae*			**1** ***C. hominis***	6 *C. fayeri*	344	2.32
O'Shannassay			9 (6 *C. ryanae*, **1** ***C. hominis***, 1 *C. suis*, **1** ***C. ubiquitum***)		1 *C. macropodum*	3 *C. macropodum*	**1** ***C. ubiquitum***	622	2.25
Silvan				**2** ***C. cuniculus***		1 *C. macropodum*		527	0.57
Tarago								61	0
Thomson								31	0
Upper Yarra		3 *C*. sp. duck genotype	4 (3 *C. ryanae*, **1** ***C. hominis***)					714	0.98
Yan Yean			2 *C. ryanae*		6 (**2** ***C. cuniculu*** *s*, 2 *C*. sp. Kangaroo genotype, 1 *C. macropodum*, 1 *C. fayeri*)			601	1.33

Each positive sample is identified to the nearest major *Cryptosporidium* clade. Overall prevalence for *Cryptosporidium* was 1.6 %. Species commonly reported in humans (26 %) are in bold-type. Bird, Reptile, Cat, Dog, Fox, Rat, Possum and Unknown groups were all test-negative for *Cryptosporidium*

In the following list, we assign individual taxa identified in the present study (based on the sequencing of *SSU*) to the most closely related species of *Cryptosporidium* in particular clades based on sequence identity:

- Members of the *C. fayeri* clade:
  Six of 74 (8.10 %, catchment MR) samples from wallabies were test-positive for *C. fayeri*. Two of 287 (0.70 %, YY) and one of 603 (0.17 %, GV) samples from kangaroos were test-positive for *Cryptosporidium* sp. EGK1 (eastern grey kangaroo type 1), two of which were novel genotypes (GenBank accession nos. KU531656 and KU531666). One of 76 (1.32 %) samples from wallabies from catchment CA was a novel genotype that was similar to *C. fayeri* (KU531658). One of the 287 (0.35 %) samples from kangaroos from catchment YY was a novel genotype similar to *Cryptosporidium* sp. kangaroo genotype I (KU531694)*.* For the first time, *C. fayeri* and *C. fayeri*-like genotypes were reported from wombats.
- Members of the *C. hominis*, *C. parvum* and *C. cuniculus* clade:
  One of 536 (0.19 %, UY) and one of 408 (0.25 %, OS) from deer were test-positive for *C. hominis*. One of 603 (0.17 %) samples from a kangaroo from catchment GV was test-positive for *C. parvum*. Five of 26 (19.2 %, GV), one of eight (12.5 %, SV) samples and one of 287 (0.35 %, YY) samples from rabbits were test-positive for *C. cuniculus*.
- Members of the *C. canis* clade:
  One of 52 (1.92 %) samples from emus from catchment CA was test-positive for *C. canis*.
- Members of the *C. suis* clade:
  One of 536 (0.19 %) samples from deer from catchment UY was test-positive for the novel *C. suis*-like genotype (KU531660).
- Members of the *C. ubiquitum* clade:
  Two of 408 (0.49 %) and one of 247 (0.40 %) samples from deer from catchments OS and CA, respectively, were test-positive for *C. ubiquitum*. Two samples from wombats were test-positive for a novel *C. ubiquitum*-like genotype.
- Members of the *C. macropodum* clade:
  Twelve of 603 (1.99 %) and one of 287 (0.35 %) of kangaroos from GV and YY, respectively, were test-positive for *C. macropodum*. Four of 53 (7.55 %) samples from wallabies from catchment OS were test-positive for *C. macropodum*-like genotypes, three of which were novel (KU531649, KU531661 and KU531679). One of 168 (0.60 %) samples from wallabies from catchment SV were test-positive for a *C. macropodum*-like genotype.
- Members of the *Cryptosporidium* sp. duck genotype clade:
  Two novel genotypes were identified from three of 55 (5.45 %) samples from waterbirds from catchment UY (KU531654 and KU531689).
- Members of the *C. ryanae* clade:
  Two of 536 (0.37 %) and one of 250 (0.40 %) samples from deer were test-positive for *Cryptosporidium* sp. deer genotype from the catchments UY and YY, respectively. One of 536 (0.19 %, UY), two of 408 (0.49 %, OS), two of 247 (0.81 %, CA) and one of 250 (0.40 %, YY) samples from deer were test-positive for the *C. ryanae*-like MW4 genotype. Two of 408 (0.49 %) samples from deer from catchment OS were test-positive for the novel genotype *C. ryanae*-like MW7 (KU531650). Two of 408 (0.49 %, OS) and one of 247 (0.40 %, CA) samples from deer were test-positive for the *C. ryanae*-like MW2 genotype.

### *Cryptosporidium* subgenotypes based on *gp60*

**Fig. 3:**
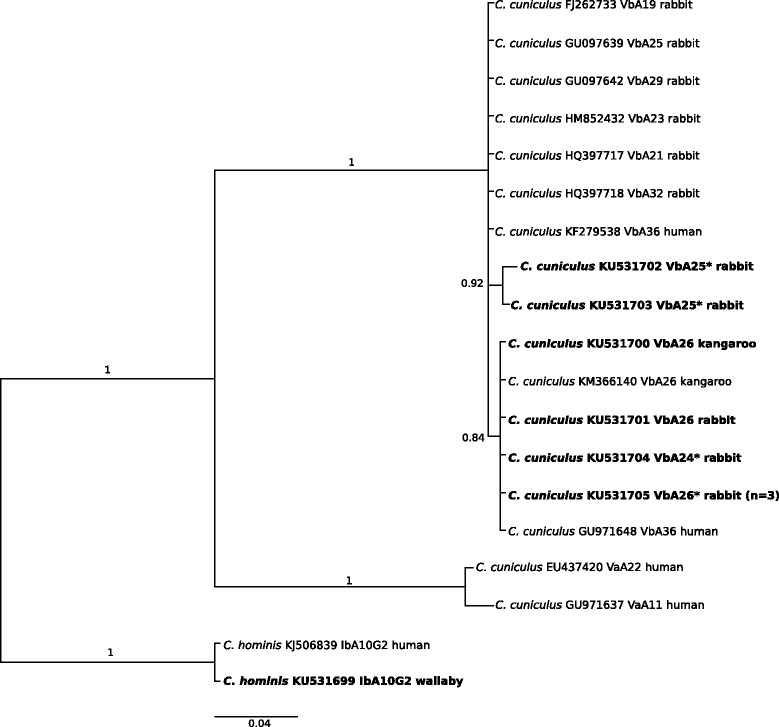
Relationships among *Cryptosporidium* taxa inferred from the phylogenetic analysis of partial 60 kDa glycoprotein gene (*gp60*) sequence data by Bayesian inference (BI). Posterior probabilities are indicated at all major nodes. Bold-type indicates *Cryptosporidium* species or genotypes characterized from faecal DNA samples in this study. In parentheses are the numbers of samples representing a particular species, genotype and sequence (GenBank accession numbers indicated). Novel genotypes (*). Scale-bar represents the number of substitutions per site

All ten samples test-positive in PCR for *gp60* (*n* = 10) were characterised to the level of subgenotype, and seven of them were assigned GenBank accession nos. (KU531699–KU531705). Based on a comparison with reference sequences from GenBank, six unique *gp60* sequence types were characterised as *C. hominis* (genotype Ib - subgenotype IbA10G2), and *C. cuniculus* (genotype Vb - subgenotype VbA25 and VbA26) (Fig. 3; Table 1 and Additional file 2: Table S5). One of 74 (1.35 %) samples from wallabies from catchment MR was test-positive for *C. hominis* IbA10G2 (accession no. KJ506839). In addition, *Cryptosporidium cuniculus* subgenotypes VbA25 and VbA26 were identified in seven samples from rabbits from catchments GV, YY, SV and MR, four of which were novel. *Cryptosporidium cuniculus* (VbA26) was identified in two samples from kangaroos from catchment YY (cf. [33]).

### *Giardia* species and assemblages

**Fig. 4:**
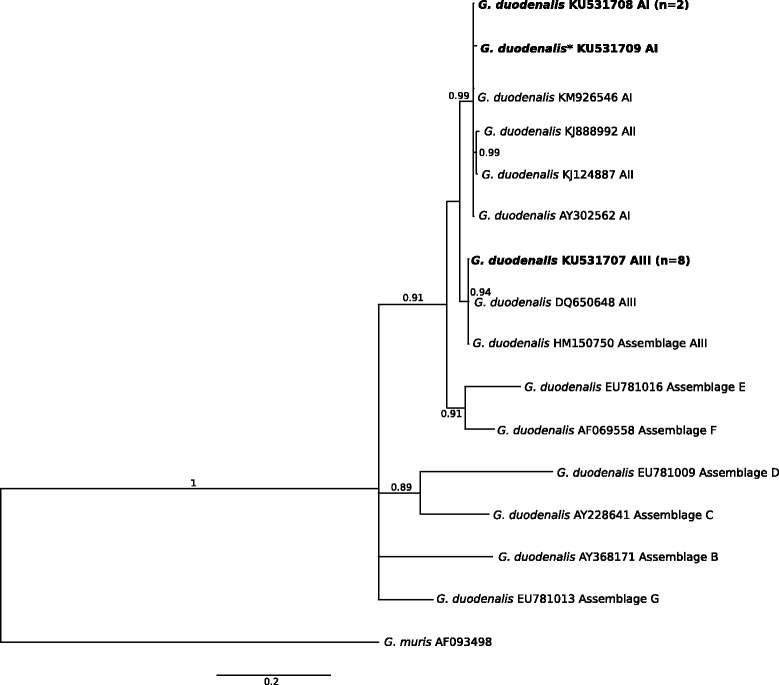
Relationships among *Giardia* taxa inferred from the phylogenetic analysis of partial triose-phosphate isomerase gene (*tpi*) sequence data by Bayesian inference (BI). Posterior probabilities are indicated at all major nodes. Bold-type indicates *Cryptosporidium* species or genotypes characterized from faecal DNA samples in this study. In parentheses are the numbers of samples representing a particular species, genotype and sequence (GenBank accession numbers indicated). Novel genotypes (*). Scale-bar represents the number of substitutions per site

Sequencing of all *tpi* amplicons identified 13 of 4,256 (0.31 %) individual faecal samples to contain *Giardia* representing the genetic assemblage A of *G. duodenalis*, based on direct sequence comparisons. From the 13 samples, we defined three distinct sequence types for *tpi* (represented by GenBank accession nos. KU531706–KU531718; Fig. 4; Tables 1 and 3 and Additional file 2: Table S6). *Giardia* sub-assemblage AI was identified in samples from a rabbit and a deer in catchment TH, a wombat in catchment MR and a kangaroo in catchment SV. The genotype (sequence type) of *Giardia* from deer identified here was novel. *Giardia* sub-assemblage AIII was identified in nine samples from deer, including five of 536 (0.93 %, UY), two of 408 (0.49 %, OS) and two of 18 (11.1 %, TAR) samples from three different catchments.

**Table 3:** The total numbers of samples from individual host animals for each catchment, as part of the Melbourne Water Corporation sampling program for waterborne pathogens (July 2011 to November 2015), together with the numbers of animals PCR test-positive for species/genotypes of *Giardia* (number of test-positive samples)

Catchment	Deer	Rabbit	Kangaroo	Wombat	Total	Prevalence (%)
Cardinia					718	0
Greenvale					638	0
Maroondah				**1(AI)**	344	0.29
O'Shannassay	2 (AIII)				622	0.32
Silvan			**1(AI)**		527	0.19
Tarago	**2 (AI)**				61	3.28
Thomson	**1 (AI)**	**1 (AI)**			31	6.45
Upper Yarra	5 (AIII)				714	0.70
Yan Yean					601	0

Sub-assemblage AI is common in wildlife and humans while sub-assemblage AIII, or fallow deer sub-assemblage, has only been found in deer. Overall prevalence for Giardia was 0.3 %. Species commonly reported in humans (46 %) are in bold-type. Bird, Emu, Waterbird, Reptile, Cat, Dog, Fox, Rat, Possum, Wallaby and Unknown groups were all test-negative for *Giardia*

## Discussion

Extending our initial monitoring program [13], the present study provides a unique perspective of the epidemiology of zoonotic protists found in wildlife inhabiting the water catchment areas of a major metropolitan city (Melbourne). Worldwide, there have been very few comprehensive wildlife surveys in catchments or watershed, with a few notable exceptions (e.g. [34–36]). In Australia, *Cryptosporidium* and/or *Giardia* have been described from catchment regions in New South Wales [37–42], Queensland [43], Victoria [13, 33, 44] and Western Australia [45].

In the first three years of the project (June 2009 to June 2011), 2,009 faecal samples were collected and tested, resulting in a prevalence of 2.8 % for *Cryptosporidium* and 3.4 % for *Giardia* [13]. In contrast, over the last four years of the project (July 2011 to November 2015), we tested more than double the number of faecal samples (*n* = 4,256), yet the prevalence of *Cryptosporidium* and *Giardia* were 1.62 % and 0.31 %, respectively. Overall, the prevalence of these protists was usually less than that reported previously in New South Wales [38, 39, 42], Victoria [13] and Western Australia [45]. The low prevalence recorded here (compared with surveys in other states of Australia) might be a consequence of testing a greater number of samples, the host groups tested, differences in local habitats and/or catchment management practices and/or proximity to agricultural land. Many factors may account for the low prevalence of *Cryptosporidium* and *Giardia* in Melbourne’s catchments over the six-year duration of the project, including animal culls, changing water levels of the reservoirs and the end of a nine-year drought [46]. One factor influencing the higher prevalence of *Giardia* in the earlier years (cf. [13]) was a hot-spot event recorded in catchment YY in April 2010, which did not recur the subsequent years. Indeed, no *Giardia* was found in catchment YY at any stage during the present investigation. Interestingly, the presence of the ungulate-specific *G. duodenalis* sub-assemblage AIII in the catchments was only recorded once between 2009 and 2011, and the first record of *Cryptosporidium* from a wombat was recorded in 2013, highlighting the rarity and ephemeral nature of these protists in this catchment system.

In the present study, we used a PCR-based approach to genetically characterise 82 samples, which were assigned to seven recognised species of *Cryptosporidium* (represented by the GenBank accession nos. KU531647–KU531705), and to the genetic assemblage ‘AI’ and ‘AIII’ of *G. duodenalis* (accession nos. KU531706–KU531718). Of the recognised species of *Cryptosporidium*, only *C. macropodum*, detected here in kangaroos, has not been reported previously from humans. The remaining six potentially zoonotic species (*C. hominis*, *C. parvum*, *C. cuniculus*, *C. ubiquitum*, *C. canis* and *C. fayeri*) were recorded from deer, emu, kangaroo, rabbit, wallaby and wombat, respectively, and from catchments CA, GV, OS, MR, SV, UY and YY (see Additional file 1: Table S1). Of the 29 species and < 40 reported genotypes of *Cryptosporidium* currently recognised [8], the causative agents of human cryptosporidiosis are typically *C. hominis* or *C. parvum* (see [47–51]); these parasites have been linked to numerous waterborne outbreaks around the world (reviewed in [52, 53]). Despite the detection of both species in catchments regions surrounding Melbourne, their low prevalence (0.07 % for *C. hominis* and 0.02 % for *C. parvum*) might suggest a low risk of waterborne transmission to humans. Nonetheless, other species and genotypes of *Cryptosporidium* may have some zoonotic significance. For example, *C. cuniculus,* with a prevalence of 0.26 %*,* was implicated in a zoonotic outbreak of cryptosporidiosis in humans in England in 2008 [54] and was linked to a number of sporadic human cases across the UK in 2007 and 2008 [55, 56], and was detected for the first time in a kangaroo in the YY catchment [33]. *Cryptosporidium ubiquitum*, detected here in deer in catchments CA and UY, might also be a concern, as it has been proposed to present a potential public health risk due to its broad geographical and host ranges, including humans in industrialised nations [49, 57, 58]. It is plausible that *C. canis* (0.02 %) and *C. fayeri* (0.26 %) might also represent minor risks to humans; *C. canis* has been detected in humans around the world [59], and *C. fayeri* was detected in a patient in New South Wales suffering from a prolonged gastrointestinal illness [60].

In the present study, we were able to assign *Cryptosporidium* to particular clades of species based on sequence identity in *SSU*, and to genotypes/subgenotypes also based on their *gp60* sequence. Within the *C. fayeri* clade, several novel genotypes characterised from samples from kangaroos were all very similar genetically to the marsupial-specific *C. fayeri*. Here, we also report the first molecularly characterized *Cryptosporidium* genotypes from wombats. To date, there is only one record of *Cryptosporidium* from a wombat [61]; however, this was not included in the review of *Cryptosporidium* of marsupials [62]. Wombat faeces have been tested for *Cryptosporidium* in multiple surveys [13, 39, 42, 63], yielding no test-positive results. Over the six years of our monitoring of Melbourne Water catchment areas ([13] and the present study), 609 faecal samples from wombats have been tested molecularly, and nine (1.48 %) were test-positive for *Cryptosporidium* and all were genetically very similar to *C. fayeri* (Fig. 2 and Additional file 2: Table S4). For the *C. hominis*, *C. parvum* and *C. cuniculus* clade, we found relatively few samples (*n* = 14) to be test-positive (by either *SSU* and/or *gp60*) for members of this clade compared with our previous study (*n* = 32) [13]. Considerably fewer samples were collected from rabbits (*n* = 97) than in the previous study (*n* = 263) [13]. The *C. hominis* positive (IbA10G2) from a sample from a wallaby from catchment MR was identical in *gp60* sequence to a novel *C. hominis* genotype found in a human in Tasmania (GenBank accession no. KJ506839; [64]), and is the first report of *C. hominis* from a swamp wallaby. The genotypes of *C. cuniculus* characterised from kangaroos have been reported recently [33]. Within the *C. canis* clade, we identified *Cryptosporidium* consistent with *C. canis* in a faecal sample from an emu, which might relate to pseudo-parasitism, whereby the parasite is ingested and passed through the gastrointestinal tract of the host without establishing an infection. Typically, emus eat plants and insects [65], which could have been contaminated with oocyst-containing faeces from feral dogs or foxes within catchment CA. Within the *C. suis* clade, we identified, for the first time, a novel *C. suis*-like genotype in a sample from a deer. *Cryptosporidium suis* has been considered to be specific to pigs, but has also been found in cattle, rodents, humans and chimpanzees [8]. *Cryptosporidium suis*-like protists that differ by only a few bases in *SSU* have been detected in cattle and rats [66, 67]. Within the *C. ubiquitum* clade, we report, for the first time, a novel *C. ubiquitum*-like genotype from wombats (Fig. 2). The samples were collected from catchments CA (July 2013) and OS (November 2014). *Cryptosporidium ubiquitum* is considered an emerging human pathogen that has been found in a wide range of wildlife, including canids, deer, primates and rodents [8]. Within the *C. macropodum* clade, we identified *C. macropodum*-like genotypes in six swamp wallabies from catchments CA, OS and SV. Three of the six genotypes were novel. Although there seems to be a differentiation within the *C. macropodum* clade between the wallabies and kangaroos in the catchments, this difference does not carry through to brush-tailed wallabies in New South Wales, where three such wallabies had a *C. macropodum* genotype and two shared *C. macropodum*-like genotypes [68]. More extensive sampling and testing of samples from wallabies and kangaroos throughout Victoria and New South Wales would be needed to clarify whether particular macropod host-affiliations exist. Within the *C. ryanae* clade, *C. ryanae* is usually found in cattle, but has also been recorded in other ruminants, such as water buffaloes and roe deer [8]. Although *C. ryanae* was not detected in this study, multiple closely related *C. ryanae*-like and *Cryptosporidium* sp. “deer” genotypes were identified in deer. The majority of the cervines in the catchments studied here are sambar deer, but it is not possible to confidently distinguish scats of sambar from those of fallow and red deer without using molecular tools. Using such tools, the specific identification of the cervine hosts would assist in assessing host affiliations and genetic diversity within the *C. ryanae* complex.

The potential role of some *Cryptosporidium* genotypes (e.g. *C. suis-*like in deer and *C. ubiquitum-*like in wombat - GenBank accession nos. KU531660 and KU531681, respectively) as zoonotic agents remains to be proven (cf. [8, 69, 70]). Further investigations of the presence and/or distribution of *Cryptosporidium* genotypes in rabbits, deer (e.g. *C. hominis*, detected here, for the first time, in wallaby) and other native and introduced wildlife in Australia, particularly in areas surrounding water catchments, are necessary, not only to determine the significance of various host groups as primary sources, potential reservoirs and amplifiers of *Cryptosporidium* for transmission to humans, but also to establish the mode(s) of transmission among reservoir animal hosts and how infection is maintained in wild animal populations [8, 62]. The present results emphasize the need for increased investigation into the true host ranges of all *Cryptosporidium* species infecting wild and domesticated animals not yet studied.

Of the currently eight recognised species of *Giardia*, *G. duodenalis* is responsible for human disease [71–73], with isolates linked to sub-assemblages AI, AII, BIII and BIV, considered to be most commonly infective to humans, whereas sub-assemblages AIII, AIV, BI and BII are recognised to be infective to animals other than humans [72, 74]. Sub-assemblage AIII, in particular, is associated with deer and other wild ungulates [72, 75, 76]. All of the three genetic variants of *tpi* detected herein represented assemblage A; two have been reported previously (e.g. GenBank accession nos. KU531708 and KU531707) (cf. [13]), and one is novel (accession no. KU531709). Since assemblages A and B of *G. duodenalis* appear to represent the greatest zoonotic risk, given their presence in humans, livestock and companion animals [71, 72, 74, 77], studying genetic variability within/among *Giardia* isolates is pivotal to inferring the zoonotic potential of distinct genotypes within this genus of parasite.

## Conclusions

The present study has provided detailed insights into the taxa of *Cryptosporidium* and *Giardia* in animals in key water catchments in Victoria. The genetic analyses indicated that 1.92 % of the 4,256 faecal samples contained *Cryptosporidium* or *Giardia* that matched species, genotypes or assemblages with the potential to infect humans. In addition, a number of new sequence records, which did not match any previously published genotypes, were identified. As nothing is known about the zoonotic potential of these new variants of *Cryptosporidium* and *Giardia*, future work should establish whether they are found in humans in Australia. Although the focus of the present study was on vast water catchment areas in south-eastern Australia, the research findings and the approach taken have considerable implications for other protected wilderness catchment areas around the world that supply unfiltered drinking water to millions of people.

## Abbreviations

AIC, Akaike information criteria; BI, Bayesian inference; *gp60*, 60 kDa glycoprotein gene; MCMC, Monte Carlo Markov Chain; ORF, open reading frame; pp, posterior probability; SSCP, single-strand confirmation polymorphism; *SSU*, small subunit of ribosomal RNA; *tpi*, triose-phosphate isomerase gene

## Additional files

Additional file 1: Table S1. Number of faecal samples examined from each host/location combination. **Table S2.** Total number of each *Cryptosporidium* genotype sampled from each catchment. **Table S3.** Total number of each *Cryptosporidium* genotype sampled from each host. (DOCX 47 kb) Additional file 2: Table S4. Pairwise comparisons of the *SSU* gene sequences among *Cryptosporidium* genotypes. **Table S5.** Pairwise comparisons of the *gp60* gene sequences among *Cryptosporidium* genotypes. **Table S6.** Pairwise comparisons of the *tpi* gene sequences among *Cryptosporidium* genotypes. (XLSX 29 kb)
