# Supplementary material for: Cryptosporidium and Giardia taxa in faecal samples from animals in catchments supplying the city of Melbourne with drinking water (2011 to 2015)
Source: Parasit Vectors. 2016 Jun 1;9:315. doi: 10.1186/s13071-016-1607-1 (PMC4888428; doi:10.1186/s13071-016-1607-1)
Supplement: Additional file 1: Table S1. — Number of faecal samples examined from each host/location combination. Table S2. Total number of each Cryptosporidium genotype sampled from each catchment. Table S3. Total number of each Cryptosporidium genotype sampled from each host. (DOCX 47 kb) [file 13071_2016_1607_MOESM1_ESM.docx]

| **Table S1** Number of faecal samples examined from each host/location combination | | | | | | | | | | | | | | | | |
| --- | --- | --- | --- | --- | --- | --- | --- | --- | --- | --- | --- | --- | --- | --- | --- | --- |
|  | **Source** | | | | | | | | | | | | | | |  |
| **Location (Abbreviation)** | **Bird** | **Emu** | **Waterbird** | **Reptile** | **Cat** | **Dog** | **Fox** | **Deer** | **Rabbit** | **Rat** | **Kangaroo** | **Possum** | **Wallaby** | **Wombat** | **Unknown** | **Totals** |
| Cardinia (CA) | 2 | 52 |  |  |  | 2 | 1 | 247 | 6 |  | 196 |  | 76 | 118 | 18 | 718 |
| Greenvale (GV) | 1 |  | 1 |  |  | 2 | 2 | 2 | 26 |  | 603 |  |  |  | 1 | 638 |
| Maroondah (MR) | 1 |  |  |  |  | 2 |  | 81 |  |  | 77 |  | 74 | 86 | 23 | 344 |
| O'Shannassay (OS) |  |  |  | 1 |  | 1 | 4 | 408 | 8 |  | 27 |  | 53 | 90 | 30 | 622 |
| Silvan (SV) | 5 | 1 |  |  |  | 5 | 1 | 1 | 8 |  | 246 |  | 168 | 70 | 22 | 527 |
| Tarago (TAR) |  |  |  |  | 1 | 4 |  | 18 | 1 |  | 16 |  |  | 21 |  | 61 |
| Thomson (TH) |  |  |  |  |  | 1 |  | 20 | 8 |  |  |  |  | 1 | 1 | 31 |
| Upper Yarra (UY) | 31 |  | 55 |  |  |  | 1 | 536 | 36 | 12 | 3 |  |  | 33 | 7 | 714 |
| Yan Yean (YY) | 5 |  |  |  |  | 1 |  | 250 | 4 |  | 287 | 3 | 17 | 16 | 18 | 601 |
| Totals | 45 | 53 | 56 | 1 | 1 | 18 | 9 | 1,563 | 97 | 12 | 1,455 | 3 | 388 | 435 | 120 | 4,256 |

| **Table S2** The total numbers of each *Cryptosporidium* genotype (using *SSU*) sampled in each catchment, as part of the Melbourne Water Corporation sampling program for waterborne pathogens (July 2011 to November 2015). Number of novel genotypes in parentheses | | | | | | | | | | | | | | | | | | | | | | | | | | | |
| --- | --- | --- | --- | --- | --- | --- | --- | --- | --- | --- | --- | --- | --- | --- | --- | --- | --- | --- | --- | --- | --- | --- | --- | --- | --- | --- | --- |
|  | |  | | |  | | |  | | | | |  | | |  | | |  | | | |  | |  |  |  |
|  | | **Cardinia** | | | **Greenvale** | | | **Maroondah** | | | | | **O'Shannassay** | | | **Silvan** | | | **Tarago** | | | | **Thomson** | | **Upper Yarra** | **Yan Yean** | **Total** |
| *Cryptosporidium canis* | | 1 | | |  | | |  | | | | |  | | |  | | |  | | | |  | |  |  | 1 |
| *Cryptosporidium cuniculus* | |  | | | 3 | | |  | | | | |  | | | 2 | | |  | | | |  | |  | 1 | 6 |
| *Cryptosporidium fayeri* | | 1 (1) | | | 1 (1) | | | 6 | | | | |  | | |  | | |  | | | |  | |  | 3 (2) | 11 |
| *Cryptosporidium hominis* | |  | | |  | | |  | | | | | 1 | | |  | | |  | | | |  | | 1 |  | 2 |
| *Cryptosporidium macropodum* | | 1 | | | 12 | | |  | | | | | 4 | | | 1 | | |  | | | |  | |  | 1 | 19 |
| *Cryptosporidium parvum* | |  | | | 1 | | |  | | | | |  | | |  | | |  | | | |  | |  |  | 1 |
| *Cryptosporidium ryanae* | | 3 | | |  | | | 1 | | | | | 6 | | |  | | |  | | | |  | | 3 | 2 | 15 |
| *Cryptosporidium* sp. duck genotype | |  | | |  | | |  | | | | |  | | |  | | |  | | | |  | | 3 (3) |  | 3 |
| *Cryptosporidium suis* | |  | | |  | | |  | | | | | 1 (1) | | |  | | |  | | | |  | |  |  | 1 |
| *Cryptosporidium ubiquitum* | | 2 (1) | | |  | | |  | | | | | 3 (1) | | |  | | |  | | | |  | |  |  | 5 |
| Total | | 8 | | | 17 | | | 7 | | | | | 15 | | | 3 | | | 0 | | | | 0 | | 7 | 7 | 65 |
| **Table S3** The total numbers of each *Cryptosporidium* genotype (using *SSU*) sampled from each host, as part of the Melbourne Water Corporation sampling program for waterborne pathogens (July 2011 to November 2015). Bird, Cat, Dog, Fox, Possum, Rat, Reptile and Unknown groups were all negative for *Cryptosporidium*. Number of novel genotypes in parentheses | | | | | | | | | | | | | | | | | | | | | |  |  |  |  |  |  |
|  |  | |  | | |  | | |  | |  | | |  | | |  | | |  | |  |  |  |  |  |  |
|  | Deer | | | Emu | | | Kangaroo | | | Rabbit | | Wallaby | | | Waterbird | | | Wombat | | | Total | | |  |  |  |  |
| *Cryptosporidium canis* |  | | | 1 | | |  | | |  | |  | | |  | | |  | | | 1 | | |  |  |  |  |
| *Cryptosporidium cuniculus* |  | | |  | | | 1 | | | 5 | |  | | |  | | |  | | | 6 | | |  |  |  |  |
| *Cryptosporidium fayeri* |  | | |  | | | 4 (3) | | |  | |  | | |  | | | 7 (1) | | | 11 | | |  |  |  |  |
| *Cryptosporidium hominis* | 2 | | |  | | |  | | |  | |  | | |  | | |  | | | 3 | | |  |  |  |  |
| *Cryptosporidium macropodum* |  | | |  | | | 14 | | |  | | 5 | | |  | | |  | | | 18 | | |  |  |  |  |
| *Cryptosporidium parvum* |  | | |  | | | 1 | | |  | |  | | |  | | |  | | | 1 | | |  |  |  |  |
| *Cryptosporidium ryanae* | 15 (1) | | |  | | |  | | |  | |  | | |  | | |  | | | 15 | | |  |  |  |  |
| *Cryptosporidium* sp. duck genotype |  | | |  | | |  | | |  | |  | | | 3 (3) | | |  | | | 3 | | |  |  |  |  |
| *Cryptosporidium suis* | 1 (1) | | |  | | |  | | |  | |  | | |  | | |  | | | 1 | | |  |  |  |  |
| *Cryptosporidium ubiquitum* | 3 | |  | | |  | | |  | |  | | |  | | | 2 (2) | | | 5 | |  |  |  |  |  |  |
| Total | 21 | | 1 | | | 20 | | | 5 | | 5 | | | 3 | | | 9 | | | 65 | |  |  |  |  |  |  |
